# Drivers of *Batrachochytrium dendrobatidis* infection load, with evidence of infection tolerance in adult male toads (*Bufo spinosus*)

**DOI:** 10.1007/s00442-023-05380-3

**Published:** 2023-05-05

**Authors:** Jaime Bosch, Barbora Thumsová, Robert Puschendorf, Jon Bielby

**Affiliations:** 1 Biodiversity Research Institute (IMIB), CSIC-University of Oviedo-Principality of Asturias, Mieres, Spain; 2 Centro de Investigación, Seguimiento y Evaluación, Parque Nacional de la Sierra de Guadarrama, Rascafría, Spain; 3 Asociación Herpetológica Española (AHE), Madrid, Spain; 4 Museo Nacional de Ciencias Naturales-CSIC, Madrid, Spain; 5 School of Biological and Marine Sciences, University of Plymouth, Plymouth, UK; 6 School of Biological and Environmental Sciences, Liverpool John Moores University, Liverpool, UK

**Keywords:** Chytridiomycosis, Capture–recapture, Body condition, Temperate areas, Reproductive effort

## Abstract

Chytridiomycosis is affecting hundreds of amphibian species worldwide, but while in tropical areas, adult individuals have been the focus of most investigations, the exact role played by infection intensity of breeding adults is not well understood in temperate areas. We conducted mark–recapture–capture surveys during spiny common toad breeding seasons from 2006 to 2018 at the site of the first recorded outbreak of chytridiomycosis in Europe, the Peñalara Massif (Sierra de Guadarrama National Park, central Spain), and collected infection samples and several variables related to the reproductive effort of male individuals. We used general linear mixed models to evaluate the contribution of study variables on the infection loads of adult male toads exhibited at their capturing date. We also analysed the differences on several male characteristics between the pond with the largest breeding population against the rest of the ponds. We found that the duration of time spent in the waterbody and the condition of the host predicted infection loads. Animals of good physical condition, that spent longer in water, have higher infection levels than individuals with the opposite set of traits. The pond supporting the largest breeding population housed smaller male toads and in poorer condition. Our results are consistent with a shift in reproductive strategy in response to infection and potentially a strategy of tolerance, rather than resistance to infection. These findings have applications for disease mitigation and theoretical implications related to the trade-offs made and the evolution of traits in response to the disease.

## Introduction

Infectious diseases have played a major role in the decline of global amphibian populations (Skerratt et al. 2007; Scheele et al. 2019). The emergence, spread, persistence, and impact of pathogens are generally driven by interactions between the pathogen, the environment, and the host. In the case of the latter, the presence in many amphibian species of a multi-stage life history is a further level of detail that has contributed greatly to the challenge of understanding the impact of emerging pathogens (Bielby et al. 2008; Hoverman et al. 2011; Valenzuela-Sánchez et al. 2021). The developmental biology of amphibians, which in many species includes a complete transition from aquatic, herbivorous larva to terrestrial carnivorous adult, means that different life-stages and age-classes can face very different risks of pathogen exposure, infection, and impact (Garner et al. 2009). Additionally, the process of metamorphosis can result in an immunological blind-spot during the transition, which may increase further the susceptibility of the host (Rollins-Smith 1998). Ultimately, species-specific responses to infection, in conjunction with their complex list histories, limit the generality of research across species (Daversa et al. 2022), populations (Walker et al. 2010), and life-stages of the same species (Gervasi et al. 2013). While some similarities in mechanism of spread and persistence (Bielby et al. 2022) may exist, these generalities in dynamics with the host are also likely to be specific to the system and certainly the pathogen in question (Rosa et al. 2017).

One of the main drivers of amphibian decline has been the emergence of the fungal pathogen *Batrachochytrium dendrobatidis* (*Bd*). This pathogen has infected thousands of species and has been linked to > 500 species declines and ~ 90 species extinctions worldwide (Scheele et al. 2019). *Bd*’s infectious stage is a mobile zoospore, which can infect keratinised areas of an amphibian’s body. However, the host may show no signs of disease until infections pass a critical threshold of infectious zoospores (the infection intensity) within an individual (e.g., Vredenburg et al. 2010). Similarly, higher infection intensities make infection more transmissible between hosts (DiRenzo et al. 2014), with associated implications for the persistence of infection within a community, the constituent parts of which may vary in their susceptibility to infection, disease, and death (Clare et al. 2016). Clearly, infection intensity is key in the spread and effects of infectious pathogens (Beldomenico and Begon 2010).

Many factors related to the host (Fernández-Beaskoetxea et al. 2016), the pathogen (Farrer et al. 2011; Rothstein et al. 2021), and the environment (Rosa et al. 2022) may interact to affect the intensity of infection and therefore the outcome of infection and disease. However, the importance of these is likely to vary both between and within species, and in particular between temperate and tropical communities. The diversity of amphibian life-histories in tropical environment includes many species that lay eggs terrestrially, or in smaller bodies of water, and in many species, those eggs may entirely forgo the larval stage and hatch as miniature versions of adults. In contrast, in temperature regions, these strategies are much less frequent, and the most common strategy is to exhibit highly seasonal, pond-breeding behaviours. This latter reproductive strategy means that individuals are likely to vary significantly in their proximity and exposure to water-bodies, and therefore *Bd,* over the course of their different life-stages and, within a life-stage, their annual activity cycle. This could have profound implications for how pathogens spread and are maintained within a population of those species. To manage infection spread in species with these life-histories and ecologies, it is therefore important to understand how infection dynamics work at each step in their life-cycle.

For temperate pond-breeding amphibians, the host–pathogen dynamics within aquatic life-stages are relatively amenable to study. As a result, we have a good foundation of knowledge of what drives infection levels in aquatic life-stages of amphibians. There is evidence that in the aquatic larval phase of anuran amphibians increasing density (Rachowicz and Briggs 2007), duration of time spent in the water (Bielby et al. 2022), lower temperatures (Fernández-Beaskoetxea et al. 2015; Bradley et al. 2019), and developmental stage (Bielby et al. 2022) are all important in dictating the intensity of infection an individual harbours. The role of post-metamorphic amphibians is typically less well understood than that of larvae (but see Wilber et al. 2022). As amphibians undergo metamorphosis, their physiology and immune system go through a radical change as well (Rollins-Smith 1998), and we know that aspects of the innate immunity, such as skin microbiome and peptides, are linked to infection outcomes (Bates et al. 2018). We also know that seasonal changes to host behaviour can alter these dynamics; post-breeding, some animals move away and can lose infection levels, or at least they drop to a level that cannot be easily detected (Daversa et al. 2018). Further, in some species, switching between habitats over the course of the life-cycle can significantly alter infection levels, suggesting that behaviour, physiology, and environment interact to cause significant changes to host–pathogen dynamics (Daversa et al. 2018). It would be useful to better understand those dynamics in the most likely scenarios of transmission for adults of pond-breeding species, that is, the breeding season, when aggregations of animals may gather at high densities in conditions most amenable to infection transmission.

Species of the genus *Bufo* have been observed as harbouring infection in different locations globally, to differing effect. At the European index site for *Bd*, the Peñalara Massif (Sierra de Guadarrama National Park, central Spain), *Bufo spinosus* (then *Bufo bufo*) were infected and experienced mass mortalities of recent metamorphosed individuals (Bosch and Martínez-Solano 2006). However, the species seemed to expand its range in response to the decline of another commonly infection species, *Alytes obstetricans*, which suffered the more severe declines both at this site (Bosch et al. 2001; Bosch and Rincón 2008), and, more generally, across its range. Despite this initial response, and the absence of die-offs of adult individuals, long-term negative effects of *Bd* have been recorded; clutch counts of *B. spinosus* have declined in the years between 1999 and 2016 in this study population (Bosch et al. 2014, 2018) and inconspicuous mass mortalities of recent metamorphosed individuals continues year to year from 1999 (Bosch et al. unpublished results) in this study population. This decline may be underpinned not only by mass mortalities of toadlets, but also by infected adult individuals having a lower survival probability, and the probability of becoming infected being higher than that of clearance of infection (Palomar et al. 2023). Combined data suggest that even in the absence of noticeable mass die-offs, there is a long-term decline in this species in response to *Bd* exposure and infection. The role played by infection intensity of breeding adults of this genus is, however, unknown.

In this field study, we aim to determine the factors influencing *Bd* infection intensity (measured by the *Bd* copy number) in adult individuals during the breeding season. Doing so will provide a foundation for better understanding the infection dynamics in adults of this species, and by extension, other aggregative, pond-breeding amphibians. This information could be useful in identifying whether adult amphibians are a possible target for focused *Bd* interventions. Specifically, we make a number of specific predictions. As in most host–pathogen systems, we predict that adults will vary in infection intensity (P1). Further, this variation will be underpinned by a number of non-mutually exclusive mechanisms: based on the importance of the larval stage in infection levels, we predict that the duration an individual remains in the water around the time of breeding will positively influence infection intensity (P2); larger animals will harbour higher infection levels (P3; Kuris et al. 1980; Bradley et al. 2019, but see Greenberg et al. 2017); an individual’s body condition will affect the level of infection that it harbours (P4). The directionality of these relationships (P3 and P4) is complex (see Sanchez et al. 2018 for a review of the subject); in some studies, larger individuals have been linked to higher infection levels as a result of them having a larger body of resources on which pathogens can feed, or larger individuals being of sufficient quality to pursue a strategy of tolerance rather than resistance to infection. In contrast, in other systems, animals in poorer condition have exhibited higher levels of infection as a result of them being less able to dedicate resources to an immune response, thereby leaving themselves more susceptible to higher levels of infection. We also predict that given *Bd*’s ability to adapt its growth rate to the environmental conditions in which it lives (Voyles et al. 2017), it is likely that infection loads will be higher when animals are sampled after a cooler period of weather (P5).

## Materials and methods

### Study site and species

Adult spiny common toads (*Bufo spinosus*) were studied at all five permanent ponds of the Peñalara Massif (Sierra de Guadarrama National Park, central Spain) where the species breed: Laguna Grande (2018 a.s.l), Laguna Chica (1956 m a.s.l), Laguna de Pájaros (2175 m a.s.l.), Charca de la Mariposa (2136 m a.s.l.), and Charca Larga y de las Piedras (2110 m a.s.l.). The surrounding terrain consists of granite outcrops and alpine grasslands at higher elevations and heathland and pine forest at lower elevations. The park is the type locality of *Bd* in Europe, with first reports of chytridiomycosis in 2001 (Bosch et al. 2001).

We conducted mark–recapture–capture surveys during toad breeding seasons (May–June) from 2006 to 2018. We walked the perimeter of each pond at night and captured toads using dipnets. Unmarked toads were tagged with passive integrated transponder (PIT tags) underneath the skin of the dorsal side, and the number of those marked individuals was recorded. We then collected a sample of skin tissue by rubbing a sterile cotton swab (ref. 300261, Deltalab Inc., Barcelona, Spain), over the ventral side of the body and thighs (20 strokes) and the webbing of the hind feet (10 strokes), consistent with standard swabbing protocols (Briggs et al. 2010). Swabs were sprayed with 95% ethanol and stored refrigerated for a few weeks until processed. Finally, all toads were weighed and their snout–vent length (SVL) was measured to the nearest mm by pressing the individual flat (ventral side) against a ruler.

### Study variables

In any year, the start of the breeding period was considered to be when the first toad was captured each year in any of the study ponds. For every captured male toad and every sampling season, we considered the following seven variables, which correspond to the questions/predictions outlined in the introduction or random effects that are important to include in the model building process:

(1) Day of arrival, defined as the first date of the breeding period on which that individual animal was captured, (2) Stay length (in days) defined as the number of days from its date of arrival to its date of sampling (the final date on which that individual was captured), (3) Sampling date, defined as the day of the season on which the *Bd* sample was taken (the first three variables correspond to P2 outlined in the introduction), (4) SVL at the sampling date (P3), (5) Body condition at the sampling date (P4), (6) Minimum air temperature registered during the 2 days preceding the sampling date in the meteorological station located less than 1.5 km away of the farthest pond (P5) (following Fernández-Beaskoetxea et al. 2015), and (7) Breeding pond used for reproduction. For variable (5) body condition, we calculated separately for sexes the residuals from a quadratic least squares regression of body mass against SVL. For variable (7) ‘breeding pond’, we coded data as one of just two levels to be included as a random effect in the models: ‘Laguna Grande’ and ‘other ponds’, because third of the study toads bred at the main pond Laguna Grande and no individuals were recorded to have moved between ponds within a breeding season or among years. To test P1—that individual toads varied in infection intensity—we compared the best models to a null model of infection intensity.

### Infection samples’ analyses

DNA was extracted using PrepMan Ultra reagent and extractions were diluted 1:10 before qPCR amplification following Boyle et al. (2004). Samples were run in duplicate and against negative and positive controls (with known concentrations of 0.1, 1, 10, and 100 genomic equivalents of zoospores, hereafter referred to as GE). A sample was assigned as positive when the infection load was equal to or higher than 0.1, and the amplification curve presented a robust sigmoidal shape. When just one replicate of a sample was amplified successfully, the sample was analysed a third time and considered positive if the curve of the third amplification represented a positive result.

### Data analysis

All continuous variables were log transformed to reach normality after adding different quantities to deal with negative or zero values (those values being 1, 3, and 22 for infection intensity, minimum air temperature, and body condition, respectively). We used partial correlation analyses to identify and discard any variables that were strongly correlated with each other. Using the remaining variables, we fitted general linear mixed models (GLMM) to partition the variance in infection intensity of toads considering six independent components of variation: inter-individual differences (with individual toads as the levels of a random factor), among ponds variability (with ‘Laguna Grande’ and ‘other ponds’ as the levels of a random factor), and four continuous independent variables that were not correlated with each other as fixed effects. These four were: stay length, SVL, body condition, and minimum air temperature. The two random factors (pond ID, and toad ID nested within pond ID) were included in every model, along with all possible combinations of one, two, or three continuous independent variables, and the full model containing both random factors and all four continuous fixed effects. Homoscedasticity and normality of residuals of the GLMMs were visually checked and did not deviate from the canonical assumptions. Models were ranked according to the corrected Akaike Information Criterion (AICc). Models within 5 AIC units of each other were considered equally well supported. The year was not included into the models, because the variable ‘minimum air temperature’ accounts the possible environmental differences across years and the random factor ‘individual’ deals with the repeated measures among years.

Finally, we calculated the daily averaged SVL and body condition, as well as the total number of captures per day, along the different breeding seasons during the study period independently for Laguna Grande and for the rest of the ponds, and used three different analyses of covariance (ANCOVA) to check the variation in body size (SVL), body condition, and number of captured males between ponds and across days. Additionally, we fitted an additional GLMM, with individual and pond as random factors, to test any change in body condition between the first and last time, an individual was captured was related to stay length. Again, these continuous variables were log transformed to reach normality after adding different quantities to deal with negative or zero values, and homoscedasticity and normality of residuals were visually checked and did not deviate from the canonical assumptions. These analyses included individuals excluded from previous analyses on the basis of their lack of *Bd* infection data.

## Results

In total, 150 *Bd* samples taken from 108 unique male toads were collected over the study period. Seventy-nine individuals were captured once, 19 captured twice, 7 captures thrice, and 3 captured four times. For each of these samples, the seven variables previously described were calculated. Forty-nine samples tested positive for *Bd* (33%). Of the 79 captured once 50 were negative, 29 positive. Of the 19 captured twice, 13 were negative both times, four positive on only one of the two captures, and two positive both times. Of the seven captured three times, one was negative on all three, five positive twice, and one positive on all three occasions. Of the three toads captured four times, one was negative on all four captures, one was positive once, and one positive on all four captures.

The variable ‘stay length’ was significantly correlated with ‘day of arrival’ and ‘sampling day’ (partial correlations: − 0.3900, *p* < 0.0001 and 0.4359, *p* < 0.0001, respectively), and ‘sampling day’ was also correlated with ‘day of arrival’ (partial correlation: 0.8750, *p* < 0.0001). Therefore, ‘day of arrival’ and ‘sampling day’ were not included in further analyses.

**Fig. 1:**
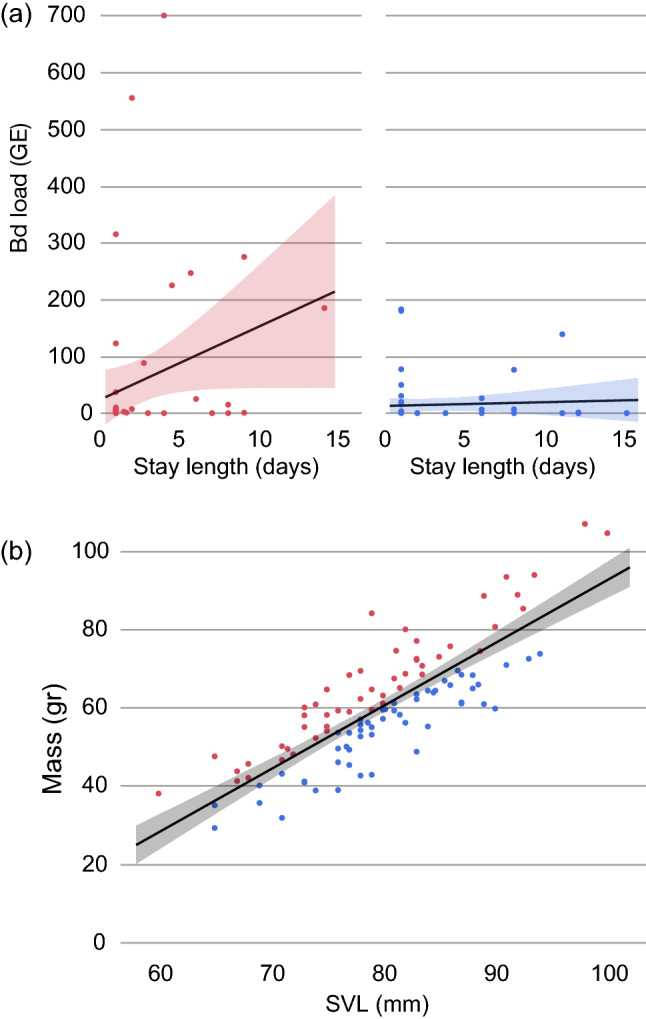
**a** Relationship between the stay length (defined as the number of days from date of arrival to date of sampling) of male *Bufo spinosus* in water for reproduction and their *Bd* infection load at the date of sampling. Red points (left panel) are averaged values (across days and seasons) of individuals with good body condition, and blue points (left panel) are for individuals with poor body condition. **b** Relationship between SVL and mass of male *B. spinosus* with good (red) and poor (blue) body condition indexes. Colored areas indicate 95% confidence intervals

The 15 different candidate GLMs appear in Table 1. Eleven out of the 15 possible models have lower AICs coefficients than the null model (AICc = 363.7). The full model appears in the fifth position, while the best model includes just the ‘length of stay’ and ‘body condition’ as explanatory variables. The second and the third best models additionally include the ‘minimum air temperature’ or ‘SVL’, respectively, and the fourth one includes just the ‘length of stay’. The first four models are within 3 AICc units of each other. The best seven performing models are within 5 AICc units of each other and all include the variable ‘length of stay’. When ‘length of stay’ and ‘body condition’ are excluded from the model, the performance drops off considerably—the best-performing model without these variables being more than 10 AICc units from the best model including it. All possible models indicate a strong effect of individual toad identity on the *Bd* infection loads (Wald’s test, *p* < 0.0147), but not pond identity (Wald’s test, *p* > 0.5295). That is, some individuals harbour higher infection intensity levels than others (P1), but the infection intensities among ponds are similar. The predictor variable contributing most to the best model is ‘stay length’ (*F*_1,118_ = 6.86, *p* = 0.0100), followed by ‘body condition’ (*F*_1,146_ = 4.97, *p* = 0.0273). In both cases, the higher values of ‘stay length’ and ‘body condition’, the higher infection intensities: males that spend more time in water for reproduction, and males showing better body condition indices tend to have higher *Bd* infections (Fig. 1). The best model parameter estimates for the length of stay in the water (0.1911) suggests that for every 1% increase in the length of stay, the *Bd* copy number increases by 1.21%. On the other hand, SVL and ‘minimum air temperature’ did not contribute significantly to any of the models.

**Table 1:** Model selection results according to the corrected Akaike Information Criterion (AICc) obtained for the *Bd* infection intensity (in logarithm) of male *Bufo spinosus* considering six independent components of variation by using general linear mixed models: inter-individual differences (with individual toads as the levels of a random factor and nested within pond), among ponds variability (with ‘Laguna Grande’ and ‘other ponds’ as the levels of a random factor), number of days spend in the pond until sampling date (stay length), snout–vent length (SVL), body condition, and minimum air temperature (MinAirT) registered during the 2 days preceding the sampling date

Continuous independent variables included	AICc	∆AICc	AICc weight
Stay length + body condition	355.1	–	0.408
Stay length + body condition + MinAirT	357.2	2.1	0.142
Stay length + body condition + SVL	357.3	2.2	0.136
Stay length	357.5	2.4	0.122
Stay length + body condition + SVL + MinAirT	359.5	4.4	0.046
Stay length + MinAirT	359.7	4.6	0.042
Stay length + SVL	359.7	4.6	0.042
Body condition	360.8	5.7	0.024
Stay length + SVL + MinAirT	361.9	6.8	0.014
Body condition + MinAirT	362.7	7.6	0.009
Body condition + SVL	362.9	7.8	0.008
Body condition + SVL + MinAirT	364.9	9.8	0.003
MinAirT	365.7	10.6	0.002
SVL	365.8	10.7	0.002
SVL + MinAirT	367.8	12.7	0.001

The AICc figure for the null model (i.e., containing just both random factors) is 363.7

**Fig. 2:**
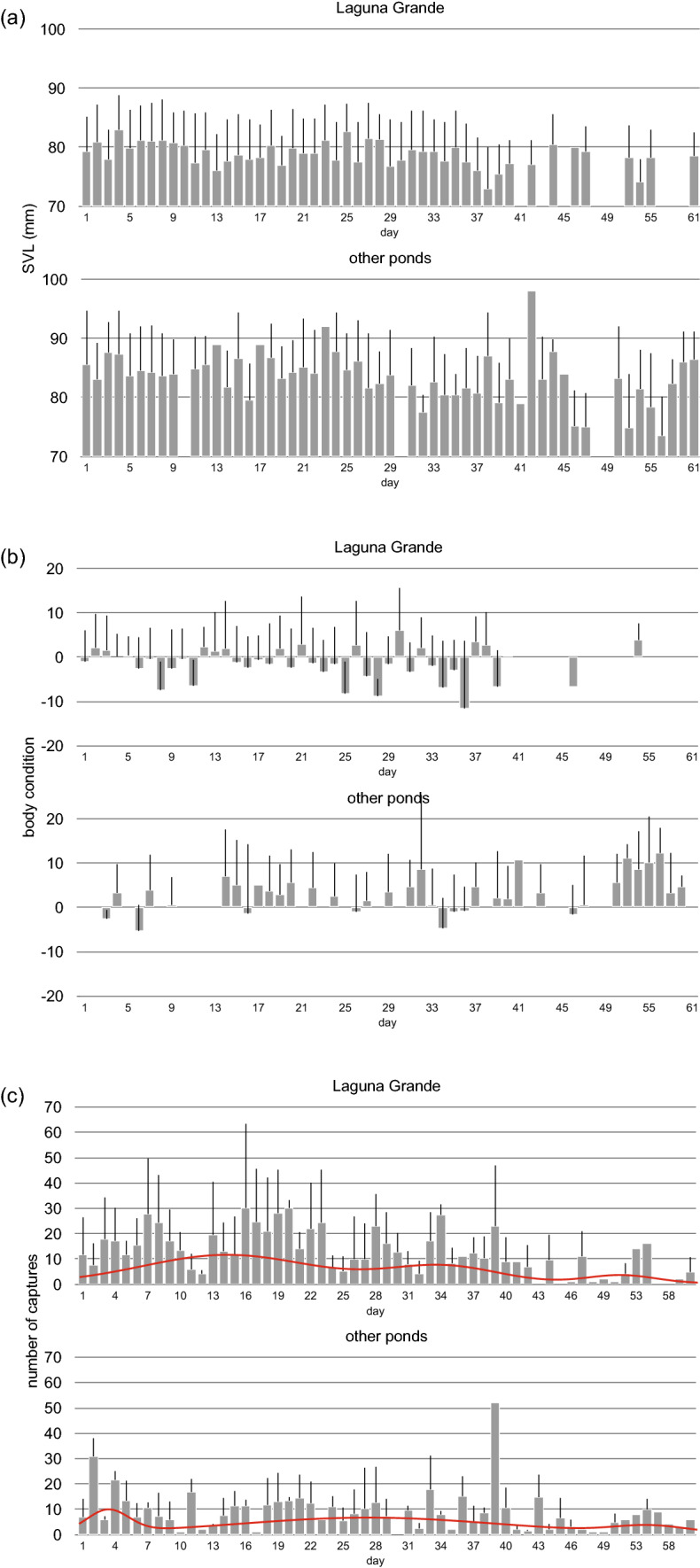
Averaged values (mean ± SE) of SVL (**a**), body condition (**b**) and number of captures (**c**) of male *Bufo spinosus* per date of the breeding period in ‘Laguna Grande’ pond and the rest of the ponds (‘other ponds’) where the species breeds at the Sierra de Guadarrama National Park in Madrid

Using the entire data set, SVL was negatively related to the day of the season (*F*_1,104_ = 6.8, *p* = 0.0107; Fig. 2A) in both groups of ponds, but was smaller at Laguna Grande that in the rest of the ponds (mean ± SE; 78.7 ± 0.52 vs. 83.3 ± 0.48; *F*_1,104_ = 41.9, *p* < 0.0001). Body condition did not change across the day of the season (*F*_1,80_ = 1.3, *p* = 0.1850; Fig. 2B) and was lower at Laguna Grande than in the other ponds (− 1.42 ± 0.63 vs. 2.85 ± 0.67; *F*_1,80_ = 21.5, *p* < 0.0001; Fig. 2B). The number of captures decreased along the breeding season (F_1,111_ = − 3.7, *p* = 0.0003; Fig. 2C) and was higher at Laguna Grande than in the rest of the ponds (13.0 ± 1.04 vs. 9.1 ± 1.00; *F*_1,111_ = 7.1, *p* = 0.0089). Finally, the change in body condition was positively related with stay length; that is, during their time in the pond males increased their mass in accordance with the duration of their stay (*F*_1,288_ = 11.5, *p* = 0.0008).

## Discussion

The infection intensity of a host may be a key predictor of both the impacts of a pathogen and the likelihood of that individual transmitting infection to others in the population. As such, it is an important epidemiological variable to measure and understand if we want to improve our knowledge of how and why infectious pathogens are transmitted and spread, as well as their population-level effects. According to these analyses, the adult stage of a widespread, abundant, temperate amphibian host suggest that individuals vary in their infection intensity (P1), and the exhibited variation is associated, to differing degrees, with the duration of time an animal spends in the water (P2) and its body condition (P4). Typically, animals of good quality, that spent longer in water, would have higher infection levels than individuals with the opposite set of traits. We discuss these findings in detail below.

The length of time an adult toad spent in the water body was the strongest predictor of the infection intensity exhibited. This variable was present in the top seven performing models, and model performance dropped off considerably in its absence. Males spending more time in water for reproduction will likely experience more contacts with environmental sources of *Bd* zoospores, with other adult *Bufo* in the pond for breeding purposes, and individuals of other species that may contribute disproportionately towards infection (in this case, overwintering *Salamandra salamandra* larvae which maintain high prevalence of infection across the whole year; Bosch et al. 2020). Combined, this higher level of infectious contacts experienced will, all else being equal, lead to a greater number of opportunities to become infected or re-infected (Rachowicz and Briggs 2007). This potentially supports the idea that in adults of pond-breeding, temperate amphibians, transmission increases as a function of density of potential sources of infection, rather than being held more evenly as host density increases (as would be the case with frequency-dependent transmission McCallum et al. 2001; Begon et al. 2002).

Our results suggest that body condition (measured by a commonly used body condition index) is a good predictor of infection intensity (body condition appeared in models 1, 2, 3, and 5). Typically, higher quality males exhibited higher infection intensities. The relationship between body condition and infection intensity is complex and not always easy to predict or understand (Beldomenico and Begon 2010). On one hand, better quality hosts may be more capable of launching stronger immune responses, making the environment more hostile for potentially infecting pathogens. If better quality hosts were more capable for launching stronger immune responses, we would simply expect higher condition hosts to exhibit lower probability of infection and lower infection load. However, empirical evidence in a range of host–pathogen systems highlight that the relationship between infection load and host condition may be underpinned by a number of mechanisms working in different ways (Sanchez et al. 2018).

Our data suggest that higher condition males were more likely to have higher *Bd* infection load. One possibility here is that higher condition individuals are better able to employ a strategy of tolerating the negative impacts of pathogen infection, rather than expending more energy by resisting or clearing it. The strategy of infection tolerance has been identified in a number of host–pathogen systems, in which it has been linked specifically to metrics of host condition (Arriero et al. 2018; Budischak et al. 2018; Kutzer et al. 2016; but see Athanasiadou et al. 2015). Here, adult males enter the water body for increased mating opportunities at the risk of pathogen exposure. Perhaps, those individuals are trading off the costs of pursuing a strategy of infection tolerance with increased mating opportunities, and only those of the best condition can do so successfully. The idea that adult *B. spinosus* are employing a strategy of tolerating infection is supported by previous research on this system, showing that adult males lose infection following post-breeding migration and are not re-infected until the following year’s return to the breeding ponds (Daversa et al. 2018). This loss of infection upon leaving the waterbody is consistent with this strategy; better condition individuals can tolerate infection, maximise their reproductive opportunities, and minimise their risk of mortality due to disease, because the non-breeding part of the cycle affords them the opportunity of clearing infection and of recuperating their energetic losses. Further, our results suggest that males tended to increase in body condition in a manner that was positively correlated with the duration of their stay in the water, although we were unable to determine whether infection intensity was coupled with this change and also varied longitudinally. The positive associations between stay duration, change in body condition, and infection intensity in our dataset are correlative, but could suggest the presence of condition-related trade-offs in breeding success and pathogen exposure strategies within this species and warrant further research.

It is also possible that fecundity compensation or terminal investment could help explain individuals in better condition having higher infection loads, and in their tendency to increase in condition. Individual-level trade-offs between reproduction and survival can lead to plasticity in phenotypes around the breeding season. For example, if a host is infected with a parasite that negatively impacts its survival or is chronic, current reproductive effort can increase, because future reproductive opportunities may be compromised (Roznik et al. 2015, Valenzuela-Sanchez et al. 2021, 2022). In our data, we may see this effect with individuals having increased their reproductive effort (in this case expressed by their body condition and the time they stay in water for reproduction) in response to an elevated infection burden, potentially at the cost of reduced survival post-breeding (Palomar et al. 2023). Previous research on bufonid species suggests that males with higher body condition may maintain higher volumes of sperm and higher fertilisation rates than individuals of lower initial condition (Hettyey et al. 2009), and may also more likely to pair with females rather than participate in breeding balls or remain single (Gastón and Vaira 2020). It is therefore possible that larger individuals are better able to pursue the tactic of a prolonged breeding season than lower condition animals. At the level of the site, male adult toads at the pond with the largest breeding population (Laguna Grande) were significantly smaller (measured by SVL) and in poorer condition (measured by body condition index) than those from other ponds. Although our data cannot identify the mechanism underlying this pattern, it is feasible that that the higher density of toads at this site would also increase competition between individuals, but as density and pathogen impacts can work synergistically, it is feasible that both are important in explained the pattern observed in our data.

In larval amphibians, water temperature is often an accurate proxy for infection load (e.g., Fernández-Beaskoetxea et al. 2015), but here, in adults, there is an apparent absence of a relationship. This fact can be explained by two reasons that are not mutually exclusive. First, air temperature cannot be perfectly correlated with water temperature during the toad breeding season, because ice is melting, lowering the water temperature. Second, during the breeding season, adult males exchange their locations from water to land surrounding the breeding ponds and, therefore, both land and water temperatures are possibly dictating the temperature of an individual male toad.

Our findings add to the body of knowledge around infection dynamics in temperate, pond-breeding amphibians. Much previous knowledge has been based around larval amphibians on account of their relative ease of sampling and study. These data add to that knowledge and identify commonalities between the life-stages of an abundant, widespread host of *Bd*. As with larvae of temperate species (Bielby et al. 2022), the duration of time spent in the waterbody predicted higher levels of infection load. In addition, we found that the condition of the host matters; individuals in better condition harboured higher infections, perhaps due to a shift in reproductive strategy (Roznik et al. 2015) or a strategy of tolerance—rather than resistance—to infection (Kutzer et al. 2016; Valenzuela-Sánchez et al. 2021). These findings have applied applications—e.g., which individuals in a pond would be priority for removal or treatment—but also theoretical implications related to the trade-offs made, the plasticity exhibited, and possibly the evolution of traits in response to *Bd* exposure and infection.

## Data Availability

The datasets analysed during the current study are available from the corresponding author on reasonable request.
